# Apomorphine

**Published:** 1900-04-21

**Authors:** 


### APOMORPHINE.

If apomorphine does not make a patient sick, what does it do? According to Dr. C. J. Douglas,[1] it acts as one of the most effectual hypnotics—prompt, safe, and sure—producing sleep in a few minutes, even though the patient may be suffering from the wildest delirium. In all sorts of cases of insomnia he finds that it is of service. About a third of the ordinary emetic dose is to be administered hypodermically, say one-thirtieth of a grain, the object being to just avoid the production of nausea and vomiting, and, if thus given, the patient is said to fall sound asleep in from five to twenty-five minutes. The direct hypnotic effect of the dose does not seem to last long—about a couple of hours, but a patient suffering from mere insomnia, without pain or other irritating cause, will often sleep on all night if sleep is once induced. Apomorphine has many advantages, one of the most useful being that an overdose cures itself by producing vomiting; and if on more extensive trial the results obtained by Dr. Douglas should be confirmed by other observers, this drug may prove of great utility as a means of wooing sleep. We confess, however, that we are somewhat sceptical. On the one hand, we do not forget the many recorded cases in which the hypodermic injection of plain water has proved effectual; and, on the other, we do not remember to have seen these remarkable hypnotic effects to follow the administration of apomorphine as an expectorant, a purpose for which it is often given to the very verge of causing emesis. The fact that sleep occurs in some cases in which spasmodic asthma is relieved by apomorphine is hardly to the point, as under such circumstances sleep follows almost any drug which gives relief. In any case, however, it is a safe thing to try.

New York Med. Jour., Mar. 17.

